# Supplementary material for: A Sox17 downstream gene Rasip1 is involved in the hematopoietic activity of intra-aortic hematopoietic clusters in the midgestation mouse embryo
Source: Inflamm Regen. 2023 Aug 8;43:41. doi: 10.1186/s41232-023-00292-4 (PMC10408172; doi:10.1186/s41232-023-00292-4)
Supplement: Supplementary file 2 — Additional file 2: Supplementary Fig. 1. A. Gel analysis of enzymatic digestion (original gel image of Fig. 1E). B. Chip assay (original gel image of Fig. 1F). Supplementary Fig. 2. A. shRasip1-IRES-GFP correlates with Rasip1 KD efficiency. shLuc- or shRasip1-transduced GFP+ cells were recovered by FACS. As indicated in the FACS profile, the light green squares show a GFP+ cell population with lower expression i.e. with the lower expression of the sh constructs. Expression level of Rasip1 in shLuc- and shRasip1-transduced cells was analyzed by RT-PCR and normalized by β-actin gene expression. RT-PCR result is in the static image that is generated by a mirror-reversal of an original across a horizontal axis, due to the sample order in the electrophoresis. B. Sorted GFP+ cells (3.0 × 10^3^) were embedded in a semisolid medium. The number of total (CFU-C) and multilineage (CFU-Mix) colonies were scored after 7 days of culture. The pair of colony numbers (sh-Luc and shRasip1) in each experimental group were plotted and connected by a line. Each symbol represents the colony numbers observed with each cell preparation from individual litters of E10.5 mice (n=4, 4 individual litters of mice from which each experimental group was set up). *p≤0.05. [file 41232_2023_292_MOESM2_ESM.pptx]

## Slide 1
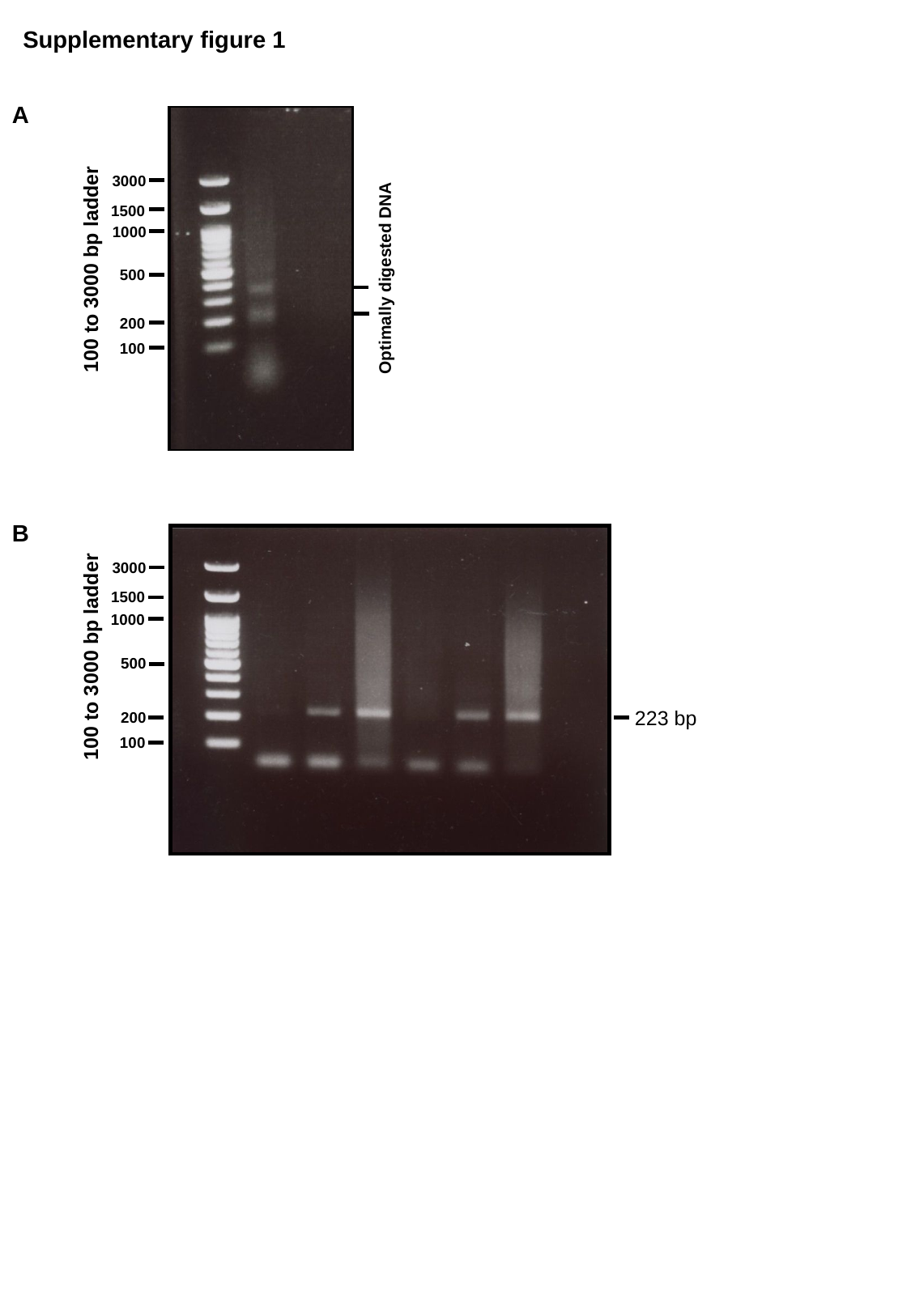

Supplementary figure 1
A
3000
1500
1000
100 to 3000 bp ladder
500
Optimally digested DNA
200
100
B
3000
1500
1000
100 to 3000 bp ladder
500
223 bp
200
100

## Slide 2
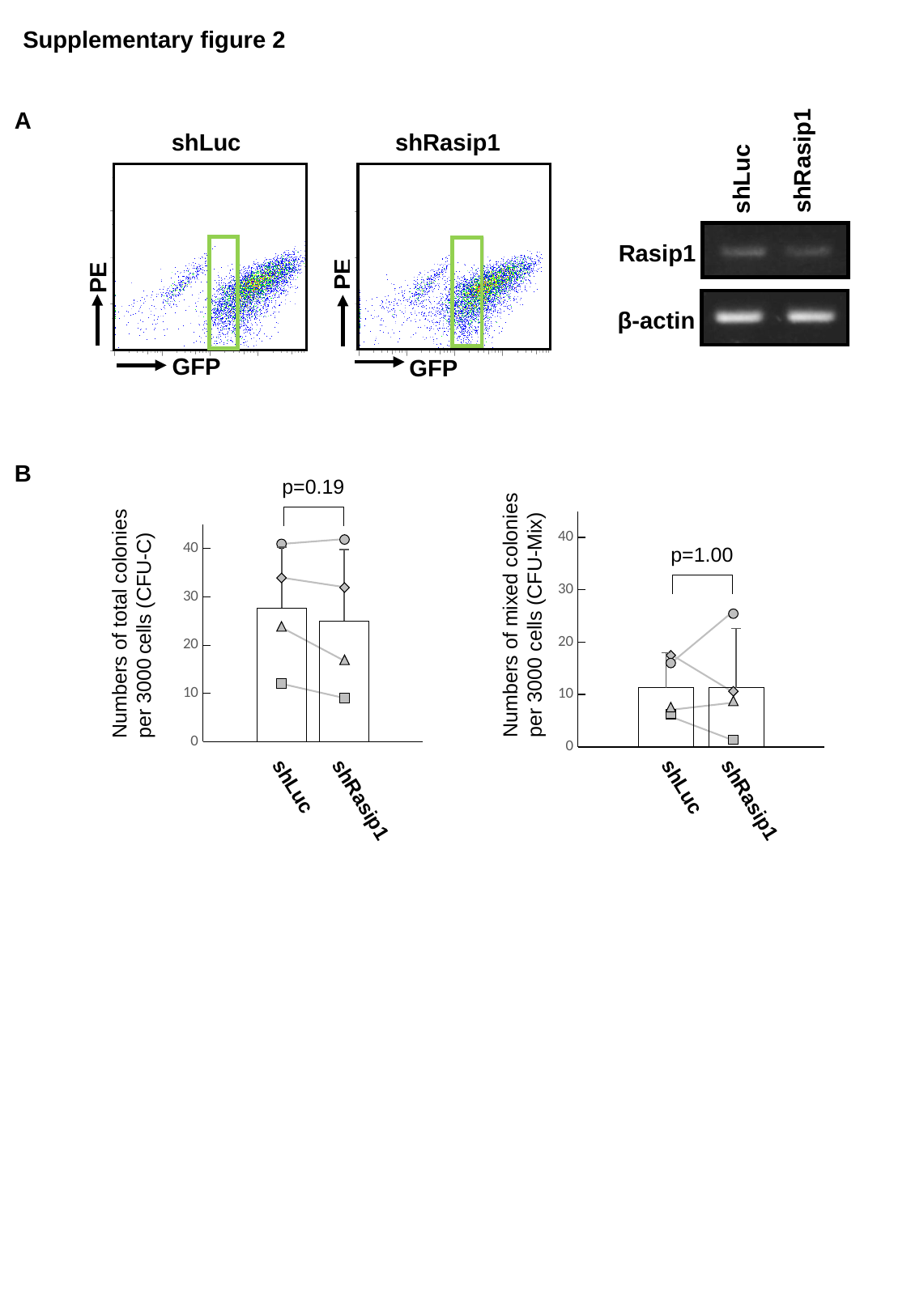

Supplementary figure 2
A
shLuc
shRasip1
shRasip1
shLuc
Rasip1
PE
PE
β-actin
GFP
GFP
B
p=0.19
Numbers of mixed colonies
per 3000 cells (CFU-Mix)
Numbers of total colonies
per 3000 cells (CFU-C)
### Chart
| Category | | |
|---|---|---|
### Chart
| Category | | |
|---|---|---|
p=1.00
shLuc
shLuc
shRasip1
shRasip1

## Slide 3
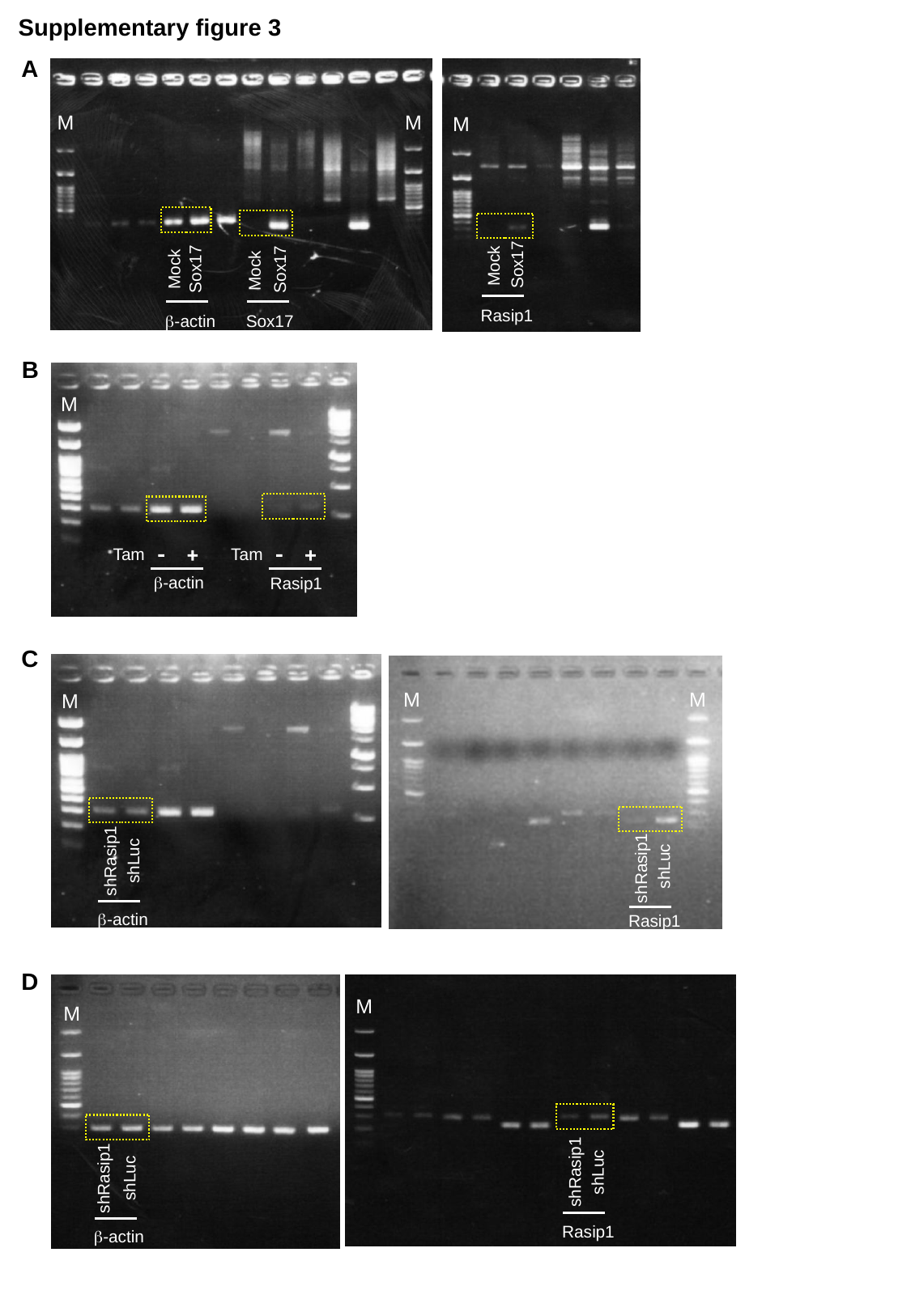

Supplementary figure 3
A
M
M
M
Sox17
Mock
Mock
Sox17
Sox17
Mock
Rasip1
b-actin
Sox17
B
M
-
+
-
+
Tam
Tam
b-actin
Rasip1
C
M
M
M
shRasip1
shLuc
shLuc
shRasip1
b-actin
Rasip1
D
M
M
shRasip1
shLuc
shRasip1
shLuc
Rasip1
b-actin

## Slide 4
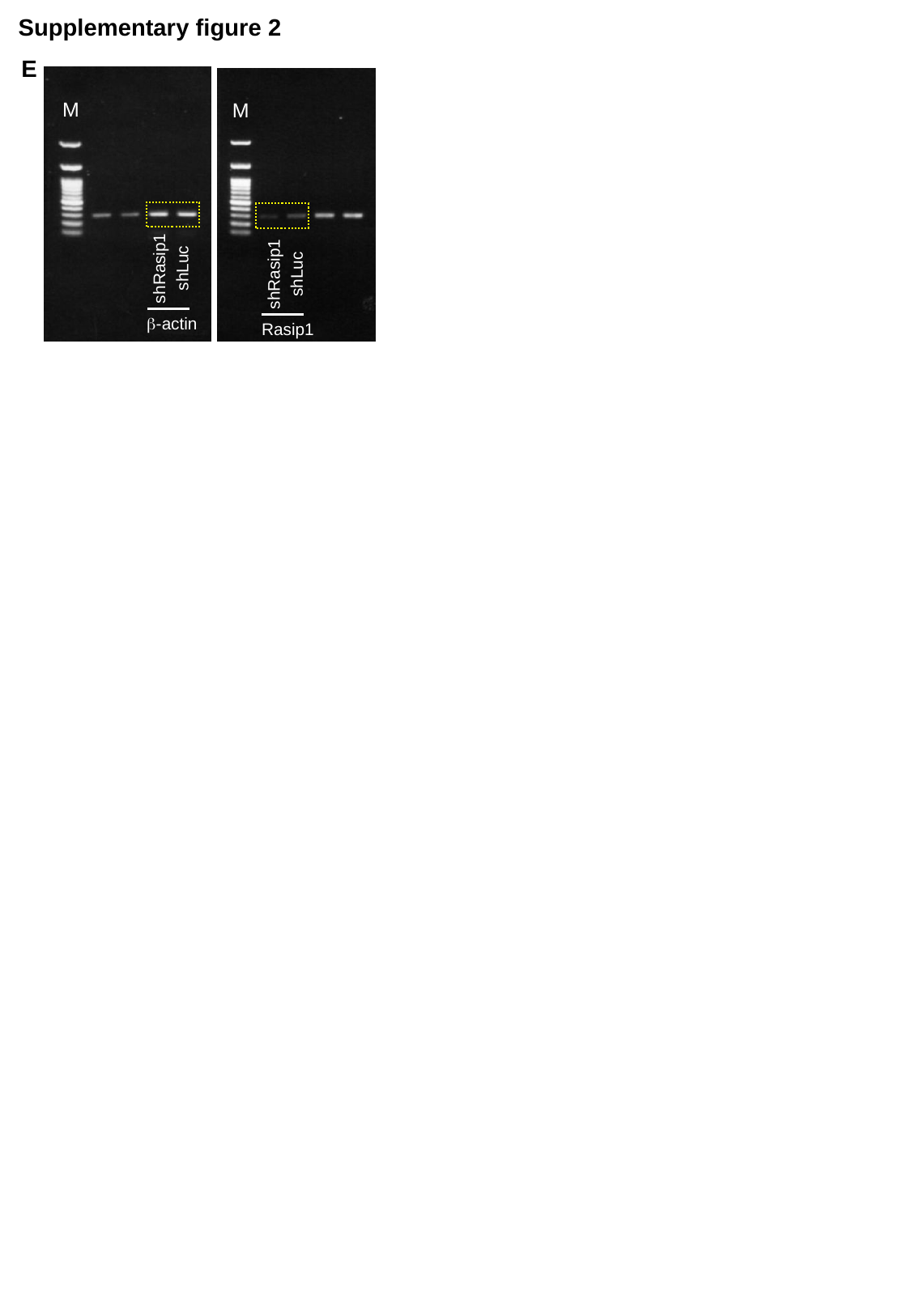

Supplementary figure 2
E
M
M
shRasip1
shLuc
shRasip1
shLuc
b-actin
Rasip1
